# The Advantages of Indocyanine Green Fluorescence Imaging in Detecting and Treating Pediatric Hepatoblastoma: A Preliminary Experience

**DOI:** 10.3389/fped.2021.635394

**Published:** 2021-02-26

**Authors:** Yu Jeong Cho, Jung-Man Namgoong, Hyun Hee Kwon, Yong Jae Kwon, Dae Yeon Kim, Seong Chul Kim

**Affiliations:** Department of Pediatric Surgery, Asan Medical Center Children's Hospital, University of Ulsan College of Medicine, Seoul, South Korea

**Keywords:** indocyanine green, hepatoblastoma, fluorescence, pediatrics, R0 resection

## Abstract

**Background:** Currently, indocyanine green (ICG) fluorescence imaging enables radical surgical resection in hepatoblastoma (HB) and has beneficial uses; however, its usage in pediatric patients is still limited.

**Methods:** From 2015 to 2019, 17 hepatoblastoma patients underwent 22 fluorescence-guided surgery using ICG. ICG (0.3 mg/kg) was intravenously injected 24–48 h before the operation. With ICG/NIR camera, intraoperative identification of biological structures and demarcation of mass were conducted.

**Results:** ICG fluorescence-guided surgery was performed for hepatoblastoma in 22 cases: 16, 1, and 2 cases underwent anatomic resection, partial hepatectomy, and liver transplantation, respectively. Six patients accompanied lung metastasis at the time of surgery, and two patients underwent lung surgery using ICG. The median interval from ICG injection to surgery was 38.3 h (range, 20.5–50.3 h). The median tumor size was 36.5 mm (range, 2–132 mm). According to the pathologic finding, the median safety margin was secured for 6 mm (range, 0–11 mm) and there was no residual finding at the liver at the follow-up computed tomography (CT).

**Conclusions:** ICG fluorescence imaging in children with HB was feasible and safe for tumor demarcation and enhancing the accuracy of radical tumor resection.

## Introduction

Chemotherapy has been used effectively to treat hepatoblastoma (HB), but complete liver resection is the gold standard treatment for this cancer. Recently, imaging modalities have improved; however, the resection range and intraoperative diagnosis of small tumors remain challenging.

Ishizawa et al. first introduced the indocyanine green (ICG) fluorescence technique in 2009 to enhance the accuracy of the liver resection procedure during hepatocellular carcinoma (HCC) surgeries (1). Protein-bound ICG emits light in the excitation spectrum at 750–810 nm, which can be visualized under near-infrared (NIR) illumination (2). ICG is extracted from the blood by normal hepatocytes and excreted into the bile. HCC cells do not excrete ICG; it is retained in HCC cells and can be detected using a camera up to several days (3). ICG imaging technique enabled highly sensitive identification of HCCs by visualizing the disordered biliary excretion of ICG.

ICG fluorescence imaging to detect HCC depended on the hepatic clearance of this compound. The pathophysiological mechanisms underlying ICG emission are not fully understood but may be associated with hepatocyte-to-bile duct secretion. It is well-established that hepatocytes take up ICG selectively taken via two membrane transport systems: OATP1B3 (transporter belonging to the family of OATP or organic anion transporters) and NTCP (Na+/taurocholate co-transporting polypeptide) (4). It was reported previously that well-differentiated HCC lesions exhibit fluorescence, whereas the tissues of poorly differentiated HCCs and colorectal tumor metastases cannot be visualized with this fluorescent marker (1). There is no study on the mechanism of action of ICG in HB; however, we can assume that HB, which is pathologically similar to HCC, can be visualized based on the disordered biliary excretion of ICG (5, 6).

We hypothesized that this imaging method could help treat HB as in HCC. Yamada et al. and Yamamichi et al. applied surgical navigation using ICG fluorescence imaging to detect HB lesions and demonstrated its usefulness in the complete resection of HB (7, 8). Other authors have reported similar results. In this single-center case series study, we evaluated the usefulness of ICG in treating pediatric HB cases, taking into account the postoperative outcomes.

## Materials and Methods

We retrospectively reviewed all children who underwent HB surgical resection assisted by ICG fluorescence imaging between January 2015 and October 2019 at Asan Medical Center, Seoul, Korea. As this is an exploratory study, statistical power was not estimated. We collected data from the medical records including patient demographics, HB characteristics such as the tumor's size and depth from the liver surface measured by preoperative contrast-enhanced computed tomography (CT), the interval time between ICG injection and surgery, type of liver resection, pattern of immunofluorescence (diffuse or uneven) pathological findings, and follow-up.

The primary endpoint was ICG imaging's feasibility in exactly demarcating HB. The secondary endpoints were disease-free survival and HB recurrence at the follow-up period. ICG was intravenously injected before surgery in each patient (0.3 mg/kg of body weight). The interval between the ICG injection and surgery was 1 or 2 days, which was set to decrease false-positive results based on our experience and other previous studies (2, 8–11). A 10 mm endoscopic NIR camera system (D-Light P, Karl Storz SE & Co. KG, Tuttlingen, Germany) was used for the intraoperative ICG fluorescence imaging. It activates molecules with emitted light at 835 nm wavelength and filters out light with a wavelength (12). In this device, the camera unit was directly handled, and intraoperative fluorescence images were observed on a monitor. Therefore, ICG-stained HB cells could be detected, being distinguishable from normal hepatocytes. Before tumor resection, the lesion area was identified by the ICG/NIR camera at 10–20 cm from the site of suspicion to demarcate the resection range. This enables us to visualize the ICG fluorescence image as it appears in the macroscopic view. The monitoring mode could be switched instantly between ICF fluorescence and normal imaging by turning on/off the ICG/NIR with one button. After resection, the fluorescence presence was evaluated again using the ICG/NIR camera in both the specimen and the resected area in real-time. Additional resection was performed if there was no remaining fluorescence in the resected area.

All the procedures described in this study were conducted following the standards of the Ethics Committee of Asan Medical Center Children's Hospital, Ulsan University (IRB No. 2019-0441).

## Results

In the time span of the study, 17 pediatric HB patients were subjected to ICG fluorescence imaging in 22 separate surgeries at our hospital. Imaging was conducted based on the patient's condition, metastatic lung tumor, liver transplantation, and anatomical resection requirements. The procedures were performed by pediatric surgeons as follows: 17 liver resections for 15 patients, 2 liver transplants (Table 1), and 3 other surgeries for lung metastases (*n* = 2) and lymph-node metastasis above the diaphragm (*n* = 1) which was done with liver resection or liver transplantation (Table 2).

**Table 1:** Clinical, pathological, surgical data of 17 children who underwent to ICG guided liver resection/transplantation for HB.

**Pt no**.	**Age (month/sex)**	**BWT at surgery**	**PRETEXT**	**Tumor diameter (cm)**	**The number of tumors (CT)**	**Metastasis**	**Invasion**	**AFP (ng/dL) at surgery**	**Interval time of ICG injection/surgery (h)**	**ICGPattern**	**Pathology/Necrosis(%)**	**Procedure**	**Safety margin (cm)**	**Disease-free survival (months)**
1	4/M	6.2	III	2.4	1			88	26.3	diffuse	Fetal/ <5	RAS	0	43
2	19/M	10.5	IV	4.7, DN*3	4	Lung	Diaphragm, Rt. adrenal gland	5,830	22.7	uneven	Epithelial and mesenchymal/95	PH (S6–8, S2/pS3)	2	41
3	18/F	9.8	I/III	6.3	1			14,800	26.3	diffuse	Fetal/0	Laparoscopic LLS	4	38
4	13/F	10	II	2.5	1			30	20.5	diffuse	Epithelial and mesenchymal/ <5	LLS	5	35
5	10/M	9.4	III	3.9	1		MHV	464	27.9	uneven	Epithelial and mesenchymal/99	CBS	0	17
6	140/F	33.9	I	13.2	1			19,199	27.0	uneven	Fetal and embryonal/10	LLS	11	12
7	43/F	9.4	IV	3.4, DN*6	7	Lung		791	23.2	diffuse	Fetal/80	RH	3	8
	46/F	10.2		4	1			55.2	39.3	uneven	Fetal and Chemotherapy-associated vasculopathy/unknown	LLS	0	
8	6/M	7.1	III	5.5	1		IVC, PV	508	45.7	undetected	Fetal/80	RTS, PH(S1)	1	7
9	16/M	9	III	5.7, DN*4	5	Lung	Diaphragm	471	37.3	uneven	Epithelial and mesenchymal/90	ERH	0.2	7
10	70/F	14.6	III	7, DN*2	3	Lung	Rt. adrenal gland	2,710	41.2	diffuse	Fetal/40	ERH, PH(S2)		4
	72/F	15.1		0.5, 1.4	3			16	40.3	diffuse	Fetal/unknown	Segmentectomy (S2, S4)	0.3	
11	5/M	6.9	III	4.8	1			207	40.0	uneven	Epithelial and mesenchymal/ <5	CBS	0	5
12	15/M	9.3	II	4.6, 4.8	2			3,060	41.2	undetected	Epithelial and mesenchymal/unknown	CBS	0.9	6
13	9/F	8	III	6.1	1		IVC	70	39.9	diffuse	Epithelial and mesenchymal/0	RH, S1	0	5
14	12/M	9.6	III	4.9	1			769	41.5	diffuse	Epithelial and mesenchymal/30	RTS	0.2	2
15	71/F	20.5	III	6.2	1	Lung		13,000	42.3	uneven	Fetal/15	LH	2.5	2
16	96/M	28	IV	13.2	Multi		Diaphragm, RHV/MHV RHV/MHV	30,500	36.7	uneven	Fetal/7	LT	none	10
17	18/F	10.1	IV	10.5	Multi	Lung		237	50.3	uneven	Epithelial and mesenchymal/30	LT	none	2

*ERH, extended right hepatectomy; R(L)H, right(left) hepatectomy; PH, partial hepatectomy; CBS, central bisegmentectomy; R(L)TS, right(left) trisegmentectomy; LTx, liver transplantation; LLS, left lateral segmentectomy; RAS, right anterior segmentectomy; DN, daughter nodule; MHV, middle hepatic vein; IVC, inferior vena cava; PV, portal vein; RHV, right hepatic vein; MHV, middle hepatic vein*.

**Table 2:** ICG fluorescence imaging of three surgeries for metastases.

**Patient no**.	**Age (months)/sex**	**BWT at surgery**	**Metastasis**	**Pathology**	**Tumor diameter (cm)**	**The number of tumors (CT)**	**AFP (ng/dL) at surgery**	**Interval time of ICG injection/surgery (h)**	**ICG pattern**	**Procedure**	**Disease-free survival (months)**
2	19/M	10.5	Lung, Rt	Non-malignant	1.5	1	5,830	22.7	diffuse	Right wedge resection	41
10	76/M	15.5	LN above diaphragm, Rt	Fetal	2.5	1	2,520	31	diffuse	Excision	4
17	18/F	10.1	Lung, Rt	Epithelial and mesenchymal	0.8, 0.9, 1	3	237	42.3	diffuse	Right wedge resection	2

None of these patients had an allergic or adverse reaction to ICG. The median age and weight of the patients at the time of their surgery were 18 months (range, 4–140 months) and 9.9 kg (6.2–33.9 kg), respectively. The median interval time between the ICG injection and surgery was 38.3 h (range, 20.5–50.3 h). The median size of the tumor was 36.5 mm (2–132 mm, except in liver transplantation cases). There were no HB lesions at a depth of more than 10 mm from the liver/lung surface, except in one case with segment II (S2) daughter nodules (Pt. No. 10) (defined as small nodular lesions around the main tumor) (13). Liver tumors at a depth of <10 mm in the liver/lung surface were clearly visible under the ICG/NIR camera. The patients were all pathologically identified as hepatoblastoma, and their subtype were as follows; epithelial and mesenchymal 9, fetal 7, fetal, and embryonal 1.

**Figure 1:**
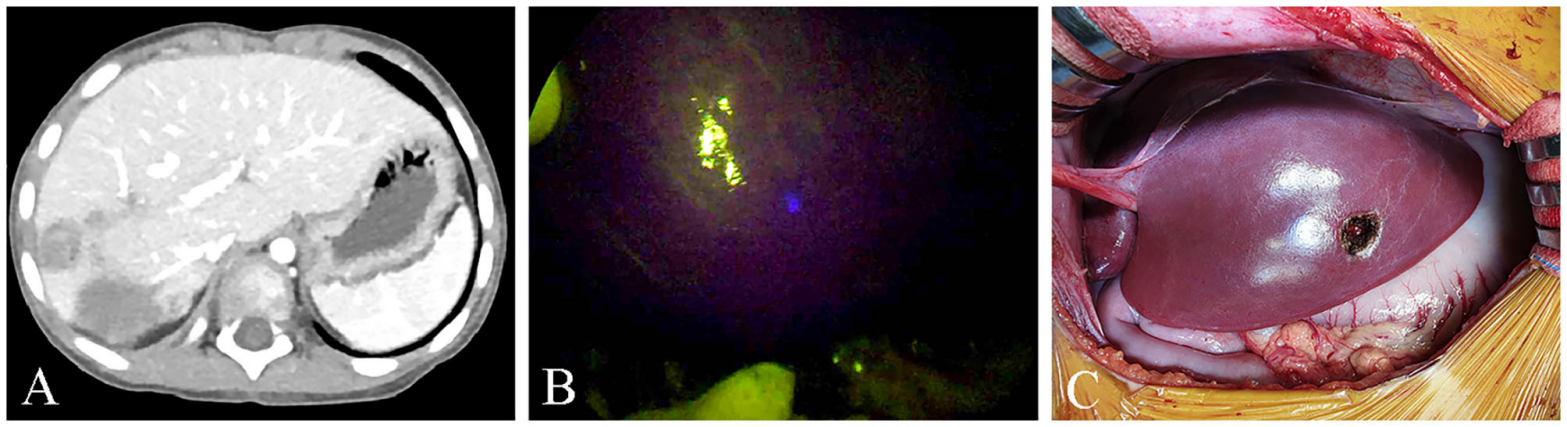
**(A,B)** One nodule was not seen on the pre-operative computed tomography (CT) but was seen on ICG/NIR camera. **(C)** Gross morphology.

In this study, 2 cases (Pt. Nos. 2 and 10) required daughter nodule resection during the main HB resection. In two patients, four nodules were removed with a median size of 10 mm on the CT image (range, 5–15 mm). Of note, one of these nodules was not detected during the preoperative examinations but revealed by the ICG/NIR camera (Figure 1), pathologically diagnosed as HB. The remaining 3 nodules had positive CT and fluorescence imaging findings but pathologically, were not HB.

In this series, two patients (Pt. Nos. 7 and 10) underwent a two-stage hepatectomy due to insufficient future liver remnant volume. In the second-stage resection in Pt. 10, S2 daughter nodules, confirmed by CT, were 15 mm from the liver surface and were not detected by the ICG/NIR camera; therefore, localization was conducted using intraoperative ultrasonography.

In two children with HB who underwent liver transplantation (Table 1), a successful inferior vena cava (IVC) demarcation was performed using ICG fluorescence imaging for safe vascular reconstruction. None of the patients who underwent primary HB resection had recurrence until now.

## Discussion

**Figure 2:**
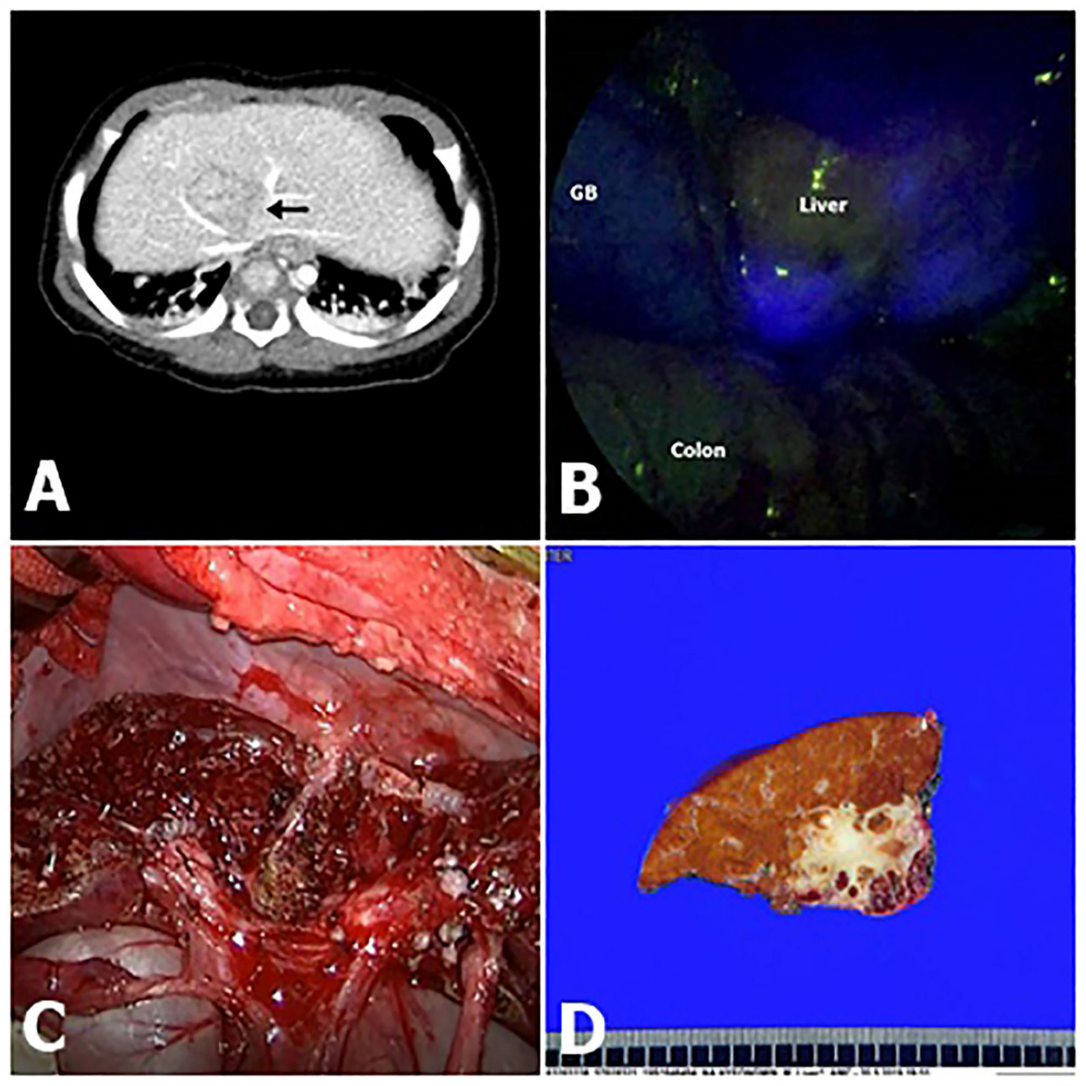
Fluorescence imaging following intravenous ICG (indocyanine green) demonstrated clear delineation (Pt. No. 11 in Table 1). **(A)** Computed tomography (CT) shows that hepatoblastoma (HB) was attached to major vessels. **(B)** Intraoperative ICG fluorescence imaging technique showed bright signals at the HB lesion with an uneven pattern. **(C)** Gross morphology. **(D)** A photograph of the liver slide of HB, showing that the resection margin is very delicate.

Up to now <50 children underwent to surgery for liver or metastatic HB with the aid of ICG fluorescence (7, 8, 10, 11, 14–17). We evaluated the potential role of intraoperative ICG fluorescence imaging in improving the radical resection of HB in a series of 17 consecutive children. ICG is a safe agent widely used to estimate liver function and assist in hepatectomy strategies for HCC and colorectal carcinoma metastases (1, 18). Our findings suggest that ICG fluorescence imaging can enable the delineation of liver segments or subsegments containing HB by visualizing the ICG retained in these lesions (Figure 2).

The major advantages of fluorescence imaging include feasibility and safety. In this case series, fluorescent images of the liver regions were obtained in real-time during the operation by placing the camera on the liver surface before resection and the raw surface during the transaction. Furthermore, only a low incidence of adverse reactions has been reported after intravenous ICG injection (19).

In this cohort, the intraoperative fluorescent finding was undetectable in Pt. Nos. 8 and 12, both with a high necrosis rate after chemotherapy (No. 8, necrosis 80%; No. 12, osseous bone differentiation/calcification). Except for these two cases, the remaining cases successfully achieved the visualization of primary HB lesions, whether in an uneven or diffuse pattern. All patients with HB underwent chemotherapy sessions before resection; therefore, a percentage of necrotic and fibrotic areas were observed macroscopically. These areas were usually non-fluorescent and had an uneven pattern (Figure 2B). Moreover, HB with teratoid features might demonstrate non-fluorescence by the same mechanism as a necrotic lesion post-chemotherapy. Ishizawa et al. demonstrated that the fluorescent patterns are closely associated with liver cancer characteristics (20). The number of subjects was small in this study; thus, there was no association between the fluorescence pattern and pathology.

Although pathophysiologic characteristics affect fluorescent signals, the liver tumor detection using the ICG technique mainly depends on the depth from the liver surface because the near-infrared light penetration depth of human tissue is limited to 5–10 mm (1, 21, 22). Kudo et al. reported that tumors located 8 mm or more from the liver surface were not identified in a series of 16 HCCs and 16 liver metastases resected from 17 patients (21). Similarly, in Pt. No. 10b, in this series, the S2 daughter nodules, evident on contrast-enhanced CT and ultrasonography, were at a 15 mm depth from the liver surface and were undetectable by ICG/NIR camera. In this case, we used intraoperative ultrasonography to localize these lesions. In patients other than Pt. No. 10b, none of the tumors were located at a depth of more than 5 mm from the liver surface which were identified using the ICG method.

**Figure 3:**
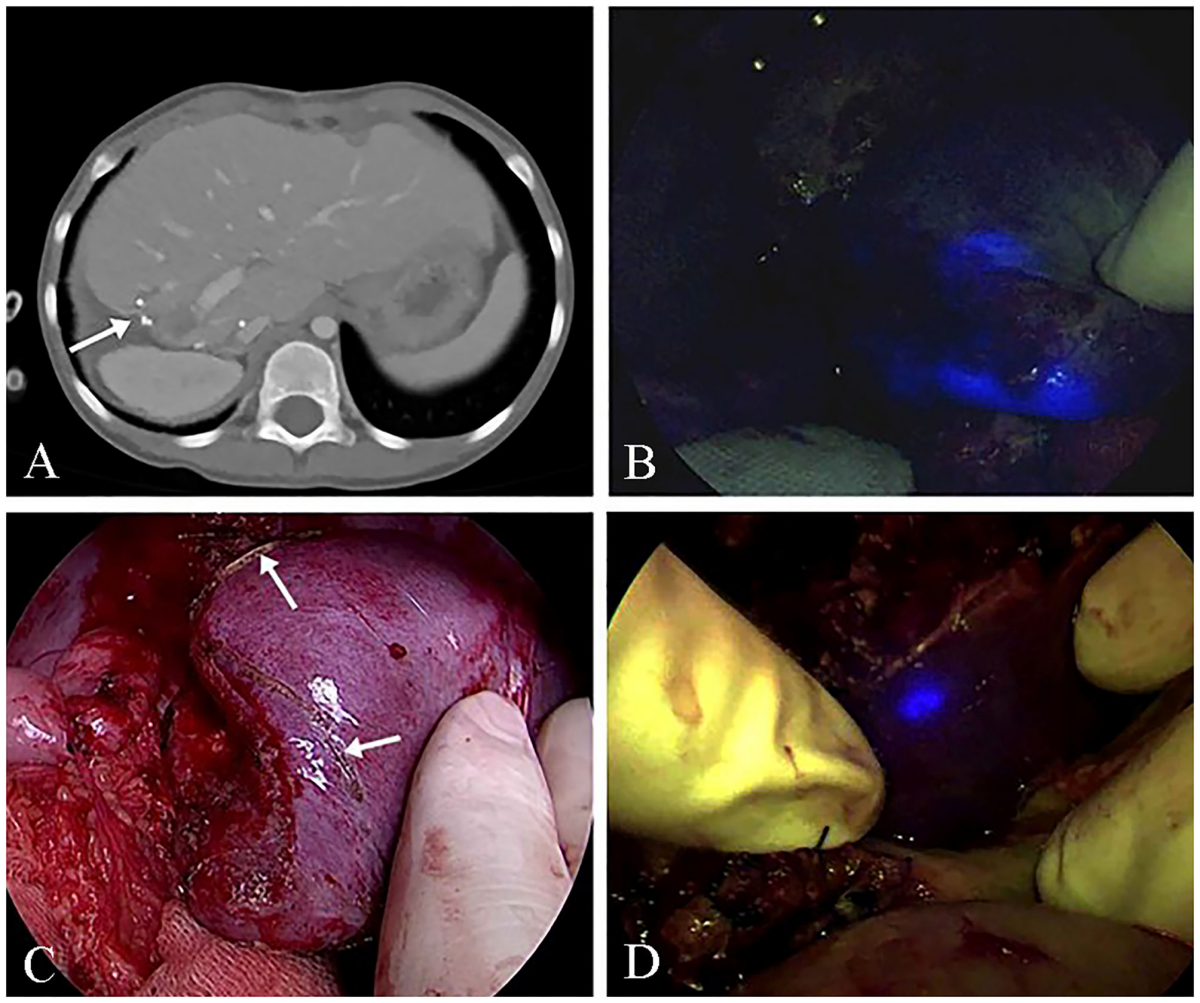
ICG (indocyanine green) fluorescence imaging of the remnant lesion at the two-stage hepatectomy (Pt. No. 10b in Table 1). **(A)** Computed tomography (CT) shows remnant HB lesions before the second-stage operation. **(B)** The intraoperative ICG fluorescence imaging technique showed the remnant HB lesions. **(C)** The delineation of the remnant HB lesion S4. **(D)** An additional resection for the fluorescence remaining in the resected area.

Despite the limitation in detecting deeply located cancers, intraoperative ICG fluorescent imaging is versatile as it is useful not only in visual inspections and palpation but also for detecting small lesions located just beneath the liver surface, compensating the drawbacks of intraoperative ultrasonography and those of laparoscopic and thoracoscopic surgery. Furthermore, this technique can detect residual cancerous tissues on the raw surface of the remnant liver after resection, which is proven to be very useful in a two-stage hepatectomy. Pt. Nos. 7 and 10 underwent a two-stage hepatectomy with prior planning for a 25–30% future liver remnant, considering the postoperative morbidity and mortality (4, 23). The lesions were checked using contrast-enhanced CT before the second surgery; however, identifying the remnant lesions boundaries on the raw surface of the remnant liver with visual or intraoperative ultrasonography during the operation is challenging. In this study, we could secure the resection margin of the lesion after the second operation (Figure 3). Regarding the oncologic aspects, this technique could achieve a more effective HB radical resection, thereby improving the outcomes (23). Besides, postoperative morbidity and mortality could be reduced by guaranteeing future liver remnant.

**Figure 4:**
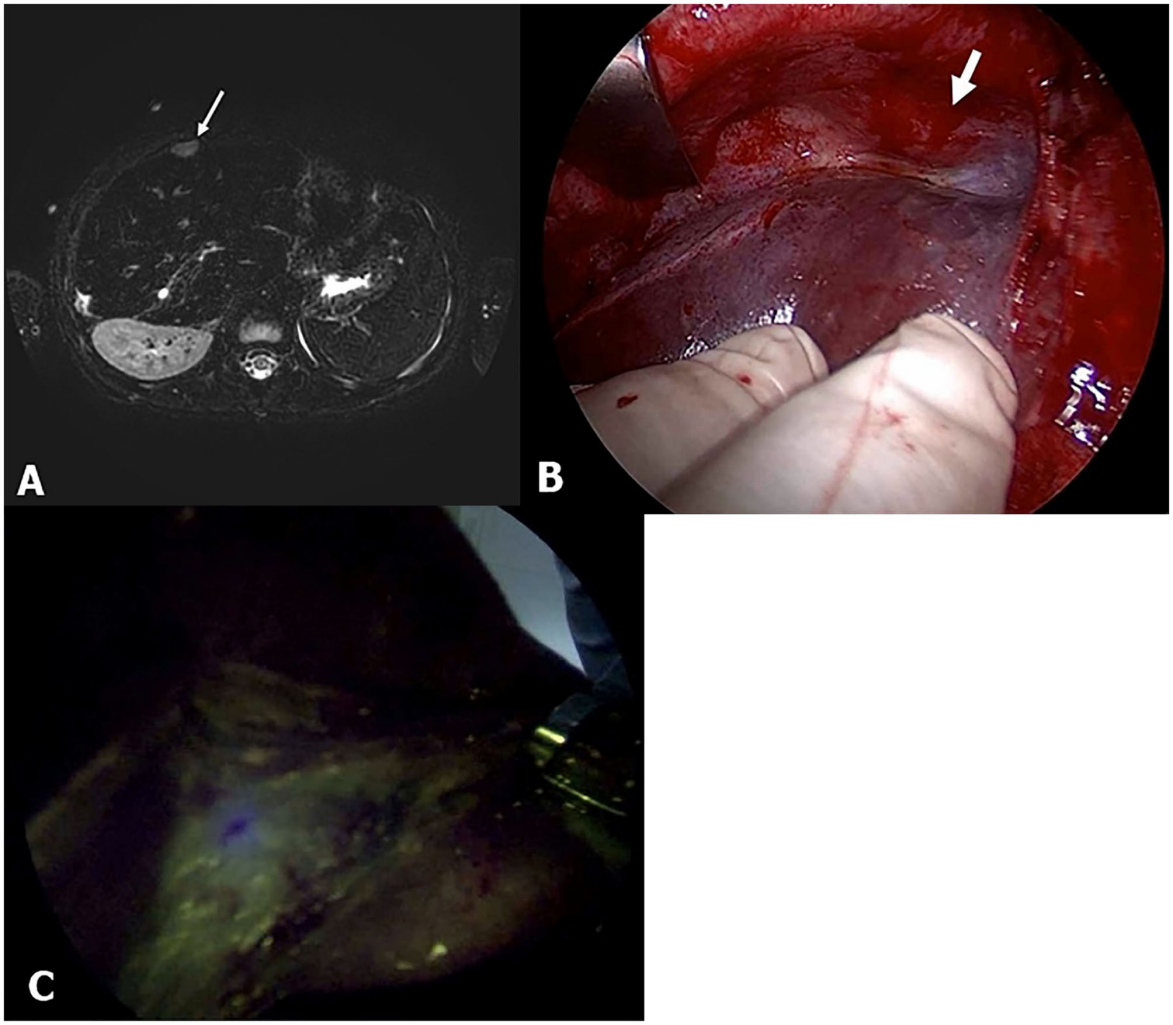
Pt. No. 10 in Table 2: **(A)** T2-weighted magnetic resonance (MR) showed the supradiaphragm metastatic lesion (white bold arrow). **(B)** Without fluorescent imaging. **(C)** Fluorescent imaging enabled visualization of the metastasis of HB that was palpable but clearly unidentified in gross.

Another advantage of the ICG technique is its ability to detect small pulmonary metastatic lesions and invade major vessels and the diaphragm adjacent to the HB (Figure 4). In this study, the pulmonary lesions were easily differentiated from the normal lung tissues (Pt. No. 2). There are several reasons why pulmonary lesions were easily detected using ICG fluorescence as follows: clear contrast created due to lack of ICG accumulation in normal lung tissue, typical metastatic lesion location in peripheral areas, and one-lung ventilation (11). The tumor cells of large HB lesions in the liver sometimes invade the IVC, major vessels, and the diaphragm. Three patients showed fluorescent signals close to the diaphragm, which, in one case (Pt. No. 16), a positive finding was confirmed requiring that part of the diaphragm to be resected en bloc with the liver. Furthermore, when a living donor liver transplantation was performed in this same patient, the ICG technology facilitated the complete resection of the HB lesion surrounding the IVC. As this method was useful in detecting any residual tumor at the resection stump of the IVC, we could determine the stump level that was safe to reconstruct anastomosis with the hepatic vein of the donor liver. Through the ICG fluorescence imaging, we could expect good liver transplantation results by securing safe vascular reconstruction.

The ICG fluorescent imaging technique has several limitations. First, the false-positive rate during surgery is reported to be 10–20% (11). In this study, eight lesions were identified on contrast-enhanced CT, ultrasonography, and fluorescent imaging. Still, they were not microscopically proved, including six daughter nodules, one metastatic lung tumor, and one invasion of the diaphragm, all <10 mm. It has been suggested that ICG is sensitive enough to detect lesions as small as 0.062 mm in diameter (11), so the resected lesions in our study were too small for pathological detection. Paraffin-embedded specimens were sectioned in μm. If the lesion is smaller than this, the pathologist could not detect it. Other possibilities of false-positive nodule might be non-specific labeling due to the poor function of non-cancerous tissue such as cirrhosis, bile duct proliferation, expanding cysts, or regenerating nodules (24). The characteristic of false-positive lesions should be clarified in further study populations in pediatrics. Second, ICG fluorescence imaging can be utilized only if the superficial lesion is <10 mm deep as the ICG signal cannot penetrate the tissues at a greater depth. It should be taken into account also that our investigation was a single-center study with small sample size, a short follow-up for the majority of our patients, and our results need to be confirmed in a larger series. Given these limitations, this technique can be used as a supplemental modality to existing imaging tests, inspections, and palpation.

## Conclusion

ICG fluorescence imaging is a promising technique that may support HB surgery as it provides additional information regarding these tumors localization and delineate the boundaries of these lesions. It might help highly sensitive identification of remnant lesions and small and grossly unidentifiable tumors in real-time. Thus, this modality can enhance the accuracy of resections and improve the oncologic results by enabling surgeons to perform R0 resection. Larger multicenter studies with long-term follow-up are warranted to confirm these findings.

## Data Availability Statement

The original contributions presented in the study are included in the article/supplementary material, further inquiries can be directed to the corresponding author.

## Ethics Statement

The studies involving human participants were reviewed and approved by the Ethics Committee of Asan Medical Center Children's Hospital, Ulsan University. Written informed consent to participate in this study was provided by the participants' legal guardian/next of kin. Written informed consent was obtained from the minor(s)' legal guardian/next of kin for the publication of any potentially identifiable images or data included in this article.

## Author Contributions

All the authors have contributed to reviewing the literature and provided a succinct account of the surgical treatment of hepatoblastoma in pediatrics.

## Conflict of Interest

The authors declare that the research was conducted in the absence of any commercial or financial relationships that could be construed as a potential conflict of interest.
